# Clinical utility of combined preimplantation genetic testing methods in couples at risk of passing on beta thalassemia/hemoglobin E disease: A retrospective review from a single center

**DOI:** 10.1371/journal.pone.0225457

**Published:** 2019-11-21

**Authors:** Chonthicha Satirapod, Matchuporn Sukprasert, Bhakbhoom Panthan, Angkana Charoenyingwattana, Pawares Chitayanan, Wasun Chantratita, Wicharn Choktanasiri, Objoon Trachoo, Suradej Hongeng

**Affiliations:** 1 Department of Obstetrics and Gynecology, Faculty of Medicine Ramathibodi Hospital, Mahidol University, Bangkok, Thailand; 2 Center for Medical Genomics, Faculty of Medicine Ramathibodi Hospital, Mahidol University, Bangkok, Thailand; 3 Panthupark Genetics Clinic, Bangkok, Thailand; 4 Department of Medicine, Faculty of Medicine Ramathibodi Hospital, Mahidol University, Bangkok, Thailand; 5 Department of Pediatrics, Faculty of Medicine Ramathibodi Hospital, Mahidol University, Bangkok, Thailand; University of Florida, UNITED STATES

## Abstract

Thalassemia and hemoglobinopathy is a group of hereditary blood disorder with diverse clinical manifestation inherited by autosomal recessive manner. The Beta thalassemia/Hemoglobin E disease (HbE/βthal) causes a variable degree of hemolysis and the most severe form of HbE/βthal disease develop a lifelong transfusion-dependent anemia. Preimplantation genetic testing (PGT) is an established procedure of embryo genetic analysis to avoid the risk of passing on this particular condition from the carrier parents to their offspring. Preimplantation genetic testing for chromosomal aneuploidy (PGT-A) also facilitates the selection of embryos without chromosomal aberration resulting in the successful embryo implantation rate. Herein, we study the clinical outcome of using combined PGT-M and PGT-A in couples at risk of passing on HbE/βthal disease. The study was performed from January 2016 to December 2017. PGT-M was developed using short tandem repeat linkage analysis around the beta globin gene cluster and direct mutation testing using primer extension-based mini-sequencing. Thereafter, we recruited 15 couples at risk of passing on HbE/βthal disease who underwent a combined total of 22 IVF cycles. PGT was performed in 106 embryos with a 3.89% allele drop-out rate. Using combined PGT-M and PGT-A methods, 80% of women obtained satisfactory genetic testing results and were able to undergo embryo transfer within the first two cycles. The successful implantation rate was 64.29%. PGT accuracy was evaluated by prenatal and postnatal genetic confirmation and 100% had a genetic status consistent with PGT results. The overall clinical outcome of successful live birth for couples at risk of producing offspring with HbE/βthal was 53.33%. Conclusively, combined PGT-M and PGT-A is a useful technology to prevent HbE/βthal disease in the offspring of recessive carriers.

## Introduction

Thalassemia is a major public health problem in Thailand where 30%–40% of the population are estimated carriers of all type of thalassemia. Beta thalassemia/Hemoglobin E disease (HbE/βthal) is the most prevalent form of thalassemia in Thailand, and large numbers of people are also affected in other Southeast Asian countries [1–4]. Beta thalassemia arises from an abnormal beta globin gene and results in decreased (β+ thalassemia) or completely absent (β° thalassemia) production of the normal beta globin chain [5]. The hemoglobin E (HbE) allele, is caused by a point mutation in codon 26 of the beta globin gene and can decrease beta globin E chains [6, 7]. The compound heterozygous condition, HbE/βthal disease, causes a surprisingly variable anemia ranging from nearly asymptomatic states to severe anemia requiring regular blood transfusion predicted by the type of mutation, i.e., β° or β+ [2, 8–10]. At present, severe thalassemia can be cured by stem cell transplantation (SCT). However, SCT is only possible for a minority of patients with a suitable human leukocyte antigen (HLA)-matched donor and who can afford the costly treatment [11, 12]. Preconception screening for thalassemia is a practice that forms part of the national public health policy of many countries and is useful for reducing the frequency of thalassemia disease in the population. In the early stages of pregnancy, prenatal diagnostic testing such as chorionic villus sampling (CVS), amniocentesis, or cord blood sampling can be offered if a couple is identified as at risk of having a child with thalassemia. Nevertheless, prenatal testing results showing that the fetus is affected may result in the decision to terminate the pregnancy and cause psychological impact to the family [13–16].

Preimplantation genetic testing (PGT) is an established procedure of embryo genetic analysis using cells obtained from embryo biopsy process. Biopsy can be performed in either cleavage-stage embryos or trophectoderm of the blastocysts. This technology has been developed to reduce the risk of genetic disease in couples who wish to identify genetic defects in embryos obtained by *in-vitro* fertilization (IVF) [17–21]. The advantage of PGT over conventional prenatal diagnosis is the avoidance of pregnancy termination. The PGT for monogenic disease (PGT-M) method, to diagnose beta thalassemia, was initially reported in 1998 and used either restriction enzyme digestion methods or denaturing gradient gel electrophoresis to perform the mutation analysis [19, 22, 23]. An important consideration to justify the use of PGT-M is the accuracy of the diagnosis obtained using the PGT technique. One of the major problems associated with PGT-M is the possibility of misdiagnosis due to allele drop out (ADO) and DNA contamination [22]. Currently, the European Society of Human Reproduction and Technology (ESHRE) recommends performing both direct and indirect mutation testing using short tandem repeat (STR) linkage analysis in PGT-M to achieve the highest accuracy rate [24, 25]. Recently, our center has demonstrated successful PGT-M for childhood neurodegenerative disease in cleavage-stage embryos using the power of linkage analysis and SNaPSHOT mini-sequencing for direct mutation detection [26].

Chromosomal aberration is a factor that contributes to the successful embryo implantation rate. Several techniques have been described to facilitate preimplantation genetic testing for aneuploidy (PGT-A) including fluorescent *in situ* hybridization (FISH), array comparative genomic hybridization (aCGH) and next-generation sequencing (NGS) [27]. In our institute, most artificial reproductive technology (ART) protocols for monogenic disorders encourage the use of combined PGT-M and PGT-A to maximize the chances of a successful pregnancy.

In this report, we presented the retrospective review of the clinical utility of PGT utilizing for couples at risk of having offspring with HbE/βthal disease obtaining reproductive service in our single center.

## Materials and methods

### Ethics statement

The study was approved by the Committee on Human Rights Related to Research Involving Human Subjects of the Faculty of Medicine Ramathibodi Hospital, ID 09-56-20. Written informed consent was performed to all participants.

### Patients

This study was a retrospective review of patients who were couples at risk of having offspring with HbE/βthal disease and requested for ART at the Faculty of Medicine Ramathibodi Hospital between January 2016 and December 2017. All couples received the similar controlled ovarian stimulation and ICSI protocol, but PGT-A protocol might differ relying on the decision of ART specialist and embryologist who were in charge at that period of time. PGT-M protocol was controlled under the supervision of single medical genetics team.

### Controlled ovarian stimulation and ICSI protocol

The antagonist protocol was started from day 2 of menstruation using 225 units of human menopausal gonadotrophins (hMG) per day (IVF-M, LG Life Sciences, Iksan-si, Korea). The gonadotropin-releasing hormone (GnRH) antagonist (Orgalutran, MSD, Ravensburg, Germany) was injected between stimulation days 7–9. Oocyte maturation was induced with human chorionic gonadotropin (hCG) treatment (IVF-C, LG Life Sciences, Iksan-si, Korea). Oocytes were picked up 36–37 hours later and intra-cytoplasmic sperm injection (ICSI) was performed within 6 hours. All embryos were doubly biopsied in trophectoderm at blastocyst stages. All embryos were cryopreserved with the vitrified method.

### Whole genome amplification (WGA)

For PGT-A, WGA were prepared in biopsied cells by PCR-based method. WGA was performed using the SurePlex^®^ DNA amplification system kit according to the manufacturer’s protocol (Illumina, San Diego CA). For PGT-M, non-PCR-based preparation was utilized. WGA was prepared by the multiple displacement amplification (MDA) method using REPLI-g mini kit (Qiagen, Hilden, Germany).

### Preimplantation genetic testing for the monogenic disorder (PGT-M)

#### Linkage analysis

Linkage analysis was performed using six STR markers focused around the *HBB* gene (Table 1; 1–6). The reaction mixture was prepared with 50-ng DNA template, 0.2 μM of each primer, 1X PCR buffer (Qiagen, Hilden Germany), 0.2 mM of each dNTP, 1X Sol Q, 1.5 mM MgCl_2_ and 0.5 U of HotStarTaq Plus DNA polymerase (Qiagen), in a final volume of 25 μl. The PCR program was as follows: (1) 95°C for 5 minutes, (2) 10 cycles of 95°C for 30 seconds, 65°C (step down temperature of 1°C every cycle so the annealing temperature in the final cycle was 55°C) for 90 seconds, and 72°C for 30 seconds, (3) 35 cycles of 95°C for 30 seconds, 55°C for 90 seconds, and 72°C for 30 seconds, and (4) a final extension at 72°C for 15 minutes (GeneAmp^®^ 2400, Applied Biosystems, Foster City, CA). PCR products were analyzed by electrophoresis on a 2% agarose gel. The fragment size of each amplicon was analyzed by capillary electrophoresis on an ABI Prism^®^ 3500 automatic DNA sequencer (Applied Biosystems, Foster City, CA) using GeneScan^®^ analysis software (Applied Biosystems). A minimum of two informative STR markers is preferred for PGT-M analysis.

**Table 1:** Summary of primers using in preimplantation genetic testing for beta thalassemia/hemoglobin E disease.

Primer's name	Nucleotide sequence (5’ to 3’)	Distance between the STR markers and *HBB* (Mb)	Tm (°C)
1. D11S4181	F-(FAM) GGGCACCTGTAATCCCA	0.478	54.4
	R- GAACTGAGACCAAGAACATTATTCC		53.7
2. D11S2351	F-(FAM)- AAGCTTCCTTCACATTCTTACAG	0.326	53.0
	R AGGAGTCACTGGATCTACTC		52.6
3. D11S1871	F-(VIC) AAGAAGTTGCCCTGATGTCT	0.119	53.9
	R- TAAAAGGAGCTGAATGCACA		52.1
4. D11S4891	F-(FAM)- GGAAATGGACCTCTGTCTC	0.002	52.3
	R- CTTTTATTCCAGCCCCAC		50.8
5. D11S1760	F-(VIC) GATCTCAAGTGTTTCCCCAC	0.136	53.2
	R- AAACGATGTCTGTCCACTCA		53.8
6. D11S1338	F-(FAM) GACGGTTTAACTGTATATCTAAGAC	0.739	50.9
	R- TAATGCTACTTATTTGGAGTGTG		50.6
7.c.2 mini-sequencing	F- AGGTACGGCTGTCATCACTT		56.0
	R- TTCATCCACGTTCACCTTGC		55.7
	F (Snapshot)- AAAAAAAAAAAAAAAAAAAAAAACTAGCAACCTCAAACAGACACCA		61.2
8.c.79 mini-sequencing	F- CCATGGTGCATCTGACTCCT		57.0
	R- GTAGACCACCAGCAGCCTAA		56.6
	F (Snapshot)- AAAAAAAAAAAAGTGAACGTGGATGAAGTTGGTGGT		61.2
9. c.316-197 mini-sequencing	F- ACAATGTATCATGCCTCTTTGC		53.7
	R-TCCAGCCTTATCCCAACCAT		56.0
	F (Snapshot)- AAAAAAAAAAAAAAAAAAAAAAACAGTGATAATTTCTGGGTTAAGG		58.7
10. c.126 mini-sequencing	F- GCACTGACTCTCTCTGCCTA		55.9
	R- CCATCACTAAAGGCACCGAG		55.6
	F (Snapshot)- AAAAAAAAAAAAAAAAAAAAAAGGTCTACCCTTGGACCCAGAGGTT		63.1
11. c.52 mini-sequencing	F- CCATGGTGCATCTGACTCCT		57.0
	R- GTAGACCACCAGCAGCCTAA		56.6
	F (Snapshot)- AAAAAAAAAAAAAAAAAAAAAATCTGCCGTTACTGCCCTGTGGGGC		65.0
12. *HBB* Amplicon 1	F- AGGTACGGCTGTCATCACTT		56.0
	R- GTTTCCCATTCTAAACTGTACCCT		54.7

#### SNaPSHOT mini-sequencing

Mini-sequencing was performed using a primer extension-based method using the SNaPshot^®^ multiplex system (Life Technologies, Carlsbad, CA). The reaction contained three oligonucleotide primers (Table 1; 7–11) for subsequent analysis by capillary electrophoresis on an ABI Prism^®^ 3500 automatic DNA sequencer (Applied Biosystems). To visualize the electrophoresis data, the peak signal was analyzed using GeneScan^®^ analysis software (Applied Biosystems).

#### Preimplantation genetic testing for aneuploidy (PGT-A)

1. **Next-generation sequencing**
  WGA was performed as described above. Results were quantified using the Qubit dsDNA HS assay kit (Life Technologies). End-repair and purified DNA for Ion Torrent 200 bp chemistry were processed before adapter ligation according to the IonXpress plus gDNA fragment library preparation protocol (Life Technologies). Gel electrophoresis was performed and the sample fraction at 330 bp was excised using E-Gel SizeSelect Gels (Life Technologies). At least five cycles of adapter mediated amplification were required to generate quantifiable sequence-ready libraries. All libraries were evaluated using the Bioanalyzer High Sensitivity Chip (Agilent Technologies, Santa Clara, CA) before clonal amplification using the Ion OneTouch system (Life Technologies). Prior to sequencing, sample enrichment was completed using the Ion OneTouch ES module. Single-end sequencing was performed following the Ion PGM Hi-Q sequencing workflow on the Ion PGM System with the use of Ion PGM sequencing 200 kit v2 (Life Technologies). Data were analyzed using the low-pass whole genome aneuploidy analysis workflow based on the hg19 genome database using Ion Reporter Cloud v.5.2 which was not capable for mosaicism analysis at that period of time (Life Technologies).
2. **Array comparative genomic hybridization (aCGH)**
  WGA and reference DNA were co-hybridized to the 24SureV3 (BlueGnome, Cambridge, UK). The reference DNA of both males and females were derived from SureRef (BlueGnome, Cambridge, UK). Genomic DNA was labeled by random priming using 1 μl of Cy3-dCTP or Cy5-dCTP (BlueGnome, Cambridge, UK), 1 μl Klenow enzyme, and 5 μl dCTP per sample. For hybridization, labeling mixes were combined and co-precipitated with Cot-1 DNA. The mixture was evaporated under centrifugation at 75 °C for 1 hour and each pellet was resuspended in 21 μl of 75°C hybridization buffer. The pellet was completely dissolved before 18 μl was spread onto the array slide and covered with a 22 × 22 mm coverslip. The slide was put in a hybridization box presoaked with 2 × saline-sodium citrate (SSC)/50% formamide and incubated at 47°C for 16 hours. After hybridization, the slides were agitated in 2 × SSC/0.05% Tween20 to remove the cover slips. The slides were then washed with 2 × SSC/0.05% Tween20 at room temperature for 10 min, washed in 1 × SSC at room temperature for 10 min, and washed in 0.1 × SSC, at 60°C for 5 min. The last wash was in 0.1 × SSC, at room temperature for 1 min before centrifugation at 170 × *g* for 3 min. After hybridization and washing, all microarray slides were scanned using Agilent SureScan (Santa Clara, CA, USA) with the Cy3 channel/photomultiplier tube setting at 450 and the Cy5 channel setting at 550. The scanner resolution was 10 μm. The image files were analyzed with BlueFuse software (BlueGnome, Cambridge, UK). All detected copy number changes were compared with known aberrations listed in publicly available databases, including ENSEMBL (Ensembl; https://uswest.ensembl.org/index.html), DECIPHER (https://decipher.sanger.ac.uk/), and the Database of Genomic Variants (DGV; http://projects.tcag.ca/variation/) using NCBI36/ng18 University of California, Santa Cruz (UCSC) assembly.

### Embryo transfer

Endometrium preparation started at day 2–3 of menstruation with 6 mg/day estradiol valerate (Bayer Schering Pharma AG, Austria) and 400 mg/day micronized progesterone (Basin Healthcare, Belgium). A single unaffected embryo was thawed and transferred to the uterus per cycle using a TDT catheter (Laboratoire CCD, Paris, France). Patients continued estradiol valerate and micronized progesterone treatment until the gestation reached 8 weeks. The medication was discontinued if the patient was not pregnant.

### Prenatal and postnatal genetic confirmation

To validate the prenatal genetic determination result, genetic confirmation was performed during the pre- and postnatal stages. Prenatal confirmation was performed at the 16^th^ gestational week by amniocentesis. Postnatal confirmation was performed on a specimen collected from cord blood after the baby was born. The biological samples were subsequently processed for DNA extraction. Confirmation of mutation in all samples was performed by PCR-genomic DNA sequencing using primer pairs spanning all detected *HBB* gene mutations (Table 1; 12–13). For sequence analysis, 20 ng of purified PCR products were sequenced by direct cycle sequencing using fluorescent-labeled dideoxy terminators (Big Dye Terminator Cycle Sequencing Ready Reaction Kit; Applied Biosystems, Foster City, CA), following the manufacturer's protocol. Sequencing reactions were run on ABI Prism^®^ 3500 automated DNA sequencer (Applied Biosystems, Foster City, CA).

## Results

### Participant characteristics

During 2016–2017, there were 15 couples who requested ART and were at risk of having offspring with HbE/βthal disease, which was approximately 5% in total couples visited our center for IVF treatment. The average maternal and paternal ages were 34.84 ± 3.56 and 36.20 ± 3.08 years, respectively. The majority of couples were healthy, with no underlying medical or gynecological conditions. Eighty percent had no currently living children. Almost half of couples had previous experience of thalassemia in their families and the others discovered their carrier status during preconception screening. About twenty-six percent had terminated pregnancy due the child being affected by HbE/βthal disease. β thalassemia mutations in the couples were diverse, including c.316-197 C>T (33.33%), c.52A>T (26.67%), c.126_129delCTTT (20%), c.2T>G (6.67%), c.92+5 G>C (6.67%), and whole gene deletion (6.67%) (Table 2).

**Table 2:** Demographic data of the participants enrolled for preimplantation genetic testing for beta thalassemia/hemoglobin E disease.

Average maternal age (years ± SD)	34.87 ± 3.56
Average paternal age (years ± SD)	36.20 ± 3.08
Underlying gynecological condition [n (%)]	
• Endometriosis	1 (6.67)
• None	14 (93.33)
Experience on previous natural conception and pregnancy	
• Yes	7 (46.67)
• No	8 (53.33)
Number of current living child(ren) [n (%)]	
• 0	12 (80)
• 1	2 (13.33)
• 2	1 (6.67)
Reasons for referral [n (%)]	
• Experience on thalassemia	7 (46.67)
• Carrier status found during preconception screening	8 (53.33)
Experience of thalassemia in the family [n (%)]	
• Recent experience on prenatal diagnosis and termination of pregnancy	4 (26.67)
• Current children affected by thalassemia disease	2 (13.33)
• Current children affected by thalassemia trait	1 (6.67)
• No experience	8 (53.33)
Type of beta thalassemia mutation [n (%)]	
• c.126_129delCTTT	3 (20)
• c.52A>T	4 (26.67)
• c.316-197 C>T	5 (33.33)
• c.2T>G	1 (6.67)
• c.92+5 G>C	1 (6.67)
• Whole gene deletion	1 (6.67)

### *In vitro* fertilization cycles and outcomes following preimplantation genetic testing

IVF cycles and outcomes following PGT-M and PGT-A are described in Table 3. The majority of couples underwent no more than two IVF cycles, except couple #005 who underwent four attempts. PGT-M of most cases was performed by linkage analysis using STR markers around the β-globin gene cluster in parallel with mini-sequencing of the mutation using the primer extension-based method. Of them, one family had PGT-M done by only linkage analysis and partial mini-sequencing on Hb E mutation without detecting *HBB* mutation since the primer extension-based method was unable to form the reaction on large gene deletion mutation (#011). Comprehensive PGT-A was performed either by microarray or next-generation sequencing-based methods based on the experience and decision of the physician in charge. To be deemed ready for transfer, embryos required PGT-M results showing that they were not disease affected and PGT-A results showing no aneuploidy. Three couples had no suitable embryos for transfer after PGT-M and PGT-A (#007, #012, and #013).

**Table 3:** Clinical and laboratory characterization of all couples obtaining assisted reproductive technology and preimplantation genetic testing for beta thalassemia/hemoglobin E disease in this study.

ID	Maternal Age (years)	Paternal Age (years)	Maternal *HBB* Mutation	Paternal *HBB* Mutation	No. of Cycle	PGT-A Choice	Outcome
001	35	33	c.126_129delCTTT	c.79G>A	1	NGS^a^	Successful pregnancy with live birth of a male baby
002	33	33	c.79G>A	c.52A>T	2	NGS	Successful pregnancy with live birth of female monozygotic twins
003	35	36	c.79G>A	c.52A>T	1	NGS	Successful pregnancy with first trimester miscarriage
004	36	37	c.316-197C>T	c.79G>A	1	NGS	Failed implantation
005	36	36	c.79G>A	c.2T>G	4	NGS	Failed implantation
006	35	37	c.92+5 G>C	c.79G>A	1	NGS	Successful pregnancy with live birth of a male baby
007	41	44	c.79G>A	c.316-197C>T	2	NGS	No embryos suitable for transfer
008	37	37	c.79G>A	c.126_129delCTTT	2	aCGH^b^	Successful pregnancy with live birth of a male baby
009	37	39	c.316-197C>T	c.79G>A	1	aCGH	Successful pregnancy with live birth of a female baby
010	38	39	c.316-197C>T	c.79G>A	1	aCGH	Failed implantation
011	32	36	c.79G>A	Deletion	1	aCGH	Successful pregnancy with live birth of male monozygotic twins
012	38	33	c.79G>A	c.126_129delCTTT	1	aCGH	No embryos suitable for transfer
013	26	34	c.316-197C>T	c.79G>A	1	NGS	No embryos suitable for transfer
014	33	37	c.52A>T	c.79G>A	1	NGS	Successful pregnancy with live birth of a female baby
015	31	32	c.79G>A	c.52A>T	2	NGS	Successful pregnancy with live birth of a female baby

^a^NGS = next-generation sequencing;

^b^aCGH = array comparative genomic hybridization

Nine of fifteen couples (60%) had successful embryo implantation and pregnancy within two IVF cycles. Unfortunately, one of them had spontaneous miscarriage in the first trimester (#003). The eight remaining couples delivered healthy babies without complication, two of the couples had monozygotic twins (#002 and #011).

### Summary of clinical outcomes after preimplantation genetic testing for beta thalassemia/hemoglobin E disease

The total number of IVF cycles was 22. Total 106 embryos were tested for PGT. The overall ADO rate was 3.89%. Combining PGT-M and PGT-A methods resulted in 80% of couples obtaining satisfactory genetic testing results. Successful genetic testing results were classified as those indicating that the embryos were non-disease affected (wild-type or carrier), did not have any chromosomal aberration, and transfer was able to occur within the first two cycles. The successful implantation rate was 64.29% among all embryos transferred. PGT accuracy of was evaluated by prenatal and postnatal genetic confirmation and the genetic status of 100% of the newborn babies was consistent with the PGT results. The overall clinical outcome of successful live birth, after the first two IVF cycles, for couple at risk of producing offspring with HbE/βthal disease in our center was 53.33% (Table 4).

**Table 4:** Summary of clinical outcome in sixteen families requested for preimplantation genetic diagnosis for beta thalassemia/hemoglobin E disease in fifteen families.

Total IVF cycles (n)	22
Embryos tested for thalassemia PGT-M [n (%)]	106 (100)
• Wild type	25 (23.58)
• Disease affected	43 (40.57)
• Carriers	28 (26.42)
• Inconclusive	4 (3.77)
• Failed whole genome amplification	6 (5.66)
Allele drop-out rate (%)	3.89
Number of couples obtaining successful genetic testing within the first 2 cycles [n (%)]	15 (100)
• Satisfactory embryo outcome	12 (80)
• Unsatisfactory embryo outcome (unable to transfer)	3 (20)
Implantation rate after embryo transfer [n (%)]	14 (100)
• Successful implantation	9 (64.29)
• Unsuccessful implantation	5 (35.71)
Accuracy of PGT by prenatal and postnatal confirmation (%)	100
Successful pregnancy after the first cycle of treatment (%)	40
Overall clinical outcome within the first 2 cycles of 15 families [n (%)]	15 (100)
• Successful pregnancy with live birth	8 (53.33)
• Successful pregnancy with first trimester miscarriage	1 (6.67)
• Failed implantation in both cycles	1 (6.67)
• Failed implantation in the first cycle and subsequent treatment cessation	2 (13.33)
• Unable to transfer in both cycles	1 (6.67)
• Unable to transfer in the first cycle and subsequent treatment cessation	2 (13.33)
Average IVF cycle number in nine women with successful pregnancy (mean ± SD)	1.33 ± 0.50

## Discussion

### Situation of preimplantation genetic testing for beta thalassemia/hemoglobin E disease in developing countries

HbE/βthal disease is a major public health problem in Thailand and other developing countries. In Thailand, the β- thalassemia carrier rate is 10% and the HbE carrier rate varies, and in many provinces in the north and northeastern parts of the country is up to 30%–50% [1, 8]. After three decades of increasing public awareness, the national policy is to perform thalassemia carrier screening in all pregnant women who obtain antenatal care services at most public hospitals [16]. The couple-at-risk will be offered prenatal diagnosis using amniocentesis or cord blood sampling to diagnose the genetic status of the fetus, which results in pregnancy termination if the fetus is affected [28, 29]. While the importance of preconception carrier screening is widely recognized, couples need to afford to pay for testing. In this analysis, half of the couples-at-risk that requested ART had thalassemia in their families, which led them to decide to undergo PGT. The other half of the couples-at-risk were people who identified as carriers during premarital or preconception screening who did not wish to take the risk of prenatal diagnosis and pregnancy termination. The option of PGT was comprehensively discussed with all couples during genetic counseling sessions with the clinical geneticist at the Genetics Clinic before starting treatment with the IVF specialist. During 2016–2017, more than 50 couples were referred to genetic counseling and, owing to the cost of treatment and the requirement to remain in central Bangkok during ovarian stimulation, 15 couples decided to perform ART-PGT. At our center, a university hospital, the cost of ICSI with combined PGT-A and PGT-M is approximately 15,000 USD, while the gross domestic product per capita in Thailand in 2017 was 6,125.70 USD (data from the World Bank Organization in 2017). Re-imbursement for PGT-M is not possible in Thailand, and other developing countries owing to national financial status and unclear regulation. Nevertheless, the cost would be lower if PGT-A is applied only in the embryos that have been found to be disease-free following PGT-M. Currently, PGT-M service is limited in Thailand. Only three medical schools mention that they are doing PGT-M for clinical research, and most PGT-M services are offered from IVF and genetics laboratories in the private sector. HbE/βthal disease is the most common consultation in each center, following by alpha thalassemia and Duchenne muscular dystrophy. In European countries, there are several centers that perform PGT-M as their main or sole activity and approximately 50 centers offer PGT-M in addition to their ART service [30]. In these centers, the most common referral is for cystic fibrosis, which is consistent with the high carrier rate in people of north European descent [25].

### Laboratory techniques used in preimplantation genetic testing for beta thalassemia/hemoglobin E disease

In our center, we developed a PGT-M protocol following ESHRE guidelines and ensure that both direct and indirect genetic testing was included in the procedure. Indirect genetic testing was achieved through by the power of STR linkage analysis [24, 31]. Relying on our in-house genetic database, we established six STR markers that were commonly informative for Thais and Southeast Asian population. The main issue with STR linkage analysis is the distance between the STR and the gene of interest and the high risk of recombination between these loci. In some particular circumstances, the STR haplotype of disease causing and non-disease-causing alleles is difficult to distinguish, making clinical prediction inconclusive. Based on the ESHRE guidelines, at least two informative STR markers are preferred for PGT-M [24, 31]. One successful factor for STR application is the number of family members recruited for linkage analysis. All cases in our series had complete family DNA samples available for linkage analysis in one of two categories: 1) both partners and previous child or fetus affected by the disease, 2) both partners and all parents from both sides. Families with a previously affected fetus that was terminated following prenatal diagnosis could request to transfer the DNA of the deceased fetus from the laboratory that performed prenatal genetic testing. This allows linkage analysis to be performed for families without enough relatives. This also allows these families to decide if they want to risk relying on direct mutation testing only, the error rate of which can be interfered with by the allele drop-out (ADO) rate. In addition to STR linkage analysis, many studies support the use of NGS-based single nucleotide polymorphism (SNP) haplotyping as a powerful tool for PGT-M [32–34].

Our primer extension-based direct mutation testing approach used the SNaPshot^®^ multiplex system, reported to be able to detect an allele fraction as low as 5% [35]. However, direct mutation testing is not appropriate for stand-alone use in the PGT-M process when there is difficulty in WGA and when the mutant allele fraction may be lower than 5% because it can result in false negative findings. Therefore, it is recommended to combine both indirect and direct genetic testing in most PGT-M approaches. Nevertheless, for some mutations, there is no need for DNA sequencing. This is illustrated by one of our participants (#011) who had a large deletion of the β- globin gene, diagnosis of which only required the reliability of indirect linkage analysis. This technical implication is also observed in other genetic conditions without the potential for mini-sequencing reactions, such as the large gene deletion observed in Duchenne muscular dystrophy and gene inversion commonly observed in Hemophilia A [36, 37].

The PGT-M process is not 100% accurate due to the chance of misinterpretation of chromosomal recombination and ADO. Therefore, all women who obtained a successful pregnancy following the PGT-M process were required to undergo prenatal diagnosis for molecular confirmation. In our center, the accuracy of PGT-M, as evaluated by prenatal and postnatal confirmation, is 100%.

### Comprehensive chromosomal aneuploidy screening, the facilitating tool for pregnancy outcome

For the families examined here, PGT-M was crucial for the select embryos unaffected by disease, and a successful implantation rate would be highly anticipated. A key factor of successful implantation is embryo chromosome status. Although non-disease embryos were transferred, chromosomal aneuploidy would be an obstacle resulting in an unsatisfactory pregnancy rate. The chromosomal aneuploidy rate is related to maternal age. Therefore, women over 35 years of age have up to a 50% chance of having an aneuploid embryo, and up to a 70% chance when the maternal age reaches 40 [38, 39]. In this study, embryo transfer was not achievable for one of 15 cases (#007). This was due to advanced maternal age of more than 40 years and only a few ova were obtained after two cycles of ovarian stimulation. Another two families (#013 and #014) had high rates of aneuploidy and the euploid embryos were unable to be transferred because their thalassemia status was unsatisfactory and inconclusive. Several studies revealed that the implantation rate after PGT-A using aCGH and NGS is up to 60 and 70%, respectively, and that the rate of successful pregnancy with live birth was 54.4%–62%. Our results showed a satisfactory implantation and a successful pregnancy with live birth rates of 64.29% and 53.33%, respectively, following PGT-A using either aCGH or NGS [40]. Hence, we have proposed to implement combined PGT-A with PGT-M at our institute to identify embryos without thalassemia disease, with the aim of achieving the highest implantation and successful pregnancy with live birth rates.

### Factors contributing in the success rate of pregnancy

There are many factors that contribute to a successful pregnancy with live birth. The successful implantation rate following the first treatment cycle in our cohort was 40% and overall success rate for pregnancy with healthy live birth was 53.33% within two IVF attempts. Despite our limited case numbers, there were no statistically significant differences (i.e., maternal age, paternal age, underlying gynecological conditions, and underlying medical conditions) between the groups that had success and those that did not. In this study, all women who achieved successful pregnancy were under the age of 38 and had no underlying gynecological conditions. One case, who achieved successful implantation but developed spontaneous miscarriage, was obese (#003). The possible failure factors in the other four cases included maternal age > 38 years (#007, #010, and #012), obesity (#004), and endometriosis (#004). Two cases had no distinctive factors (#005 and #013). Advanced maternal age and endometriosis impact fertility [41–45]. Obesity is a detrimental factor for IVF outcome and requires a significant higher dose of gonadotropins and longer ovarian stimulation duration [46].

### Other applications of preimplantation genetic testing for beta thalassemia/Hb E disease

HbE/βthal disease is a hereditary blood disorder that can be cured by SCT. Initially, SCT requires a donor with a Human Leukocyte Antigen (HLA) profile that is matched to the recipient [47]. In families that have child(ren) affected by HbE/βthal disease and are awaiting SCT, PGT-M can help in the selection of embryos with HLA genetics consistent with the affected child using STR linkage analysis around the HLA gene region on chromosome 6. Moreover, there have been reports of the use of NGS in preimplantation high-resolution HLA sequencing [48].

### Conclusion

HbE/βthal disease is a common serious medical condition prevalent in many developing countries. PGT can be used by the couple-at-risk to reduce the chance of passing deleterious mutations onto their offspring which prevents the burden in the family. We propose a strategy of combining PGT-M and PGT-A to achieve the highest chance for successful implantation and pregnancy. Application of STR linkage analysis and direct mutation testing is standard practice for PGT-M, but chromosomal recombination and ADO may lead to misinterpretation of results. Therefore, a robust PGT-M protocol should be able to detect ADO and recombination and result in higher accuracy. PGT-A, using either aCGH or NGS, is helpful to select euploid embryos and increase the rate of successful implantation.

## References

[1] Panich V, Pornpatkul M, Sriroongrueng W. The problem of thalassemia in Thailand. Southeast Asian J Trop Med Public Health. 1992;23 Suppl 2:1–6. . 1298980

[2] Origa R. Beta-Thalassemia In: Adam MP, Ardinger HH, Pagon RA, Wallace SE, Bean LJH, Stephens K, et al, editors. GeneReviews((R)). Seattle (WA) 1993.

[3] Weatherall DJ, Williams TN, Allen SJ, O'Donnell A. The population genetics and dynamics of the thalassemias. Hematol Oncol Clin North Am. 2010;24(6):1021–31. 10.1016/j.hoc.2010.08.010 . 21075278

[4] Weatherall DJ. The Evolving Spectrum of the Epidemiology of Thalassemia. Hematol Oncol Clin North Am. 2018;32(2):165–75. 10.1016/j.hoc.2017.11.008 . 29458724

[5] Thein SL. The molecular basis of beta-thalassemia. Cold Spring Harb Perspect Med. 2013;3(5):a011700 10.1101/cshperspect.a011700 . 23637309 PMC3633182

[6] Orkin SH, Kazazian HH Jr., Antonarakis SE, Ostrer H, Goff SC, Sexton JP. Abnormal RNA processing due to the exon mutation of beta E-globin gene. Nature. 1982;300(5894):768–9. 10.1038/300768a0 . 7177196

[7] Traeger J, Wood WG, Clegg JB, Weatherall DJ. Defective synthesis of HbE is due to reduced levels of beta E mRNA. Nature. 1980;288(5790):497–9. 10.1038/288497a0 . 7442796

[8] Fucharoen S, Winichagoon P, Pootrakul P, Piankijagum A, Wasi P. Variable severity of Southeast Asian beta 0-thalassemia/Hb E disease. Birth Defects Orig Artic Ser. 1987;23(5A):241–8. . 3689905

[9] Fucharoen S, Ketvichit P, Pootrakul P, Siritanaratkul N, Piankijagum A, Wasi P. Clinical manifestation of beta-thalassemia/hemoglobin E disease. J Pediatr Hematol Oncol. 2000;22(6):552–7. 10.1097/00043426-200011000-00022 . 11132229

[10] Fucharoen S, Winichagoon P. Clinical and hematologic aspects of hemoglobin E beta-thalassemia. Curr Opin Hematol. 2000;7(2):106–12. 10.1097/00062752-200003000-00006 . 10698297

[11] Lucarelli G, Isgro A, Sodani P, Gaziev J. Hematopoietic stem cell transplantation in thalassemia and sickle cell anemia. Cold Spring Harb Perspect Med. 2012;2(5):a011825 10.1101/cshperspect.a011825 . 22553502 PMC3331690

[12] Elborai Y, Uwumugambi A, Lehmann L. Hematopoietic stem cell transplantation for thalassemia. Immunotherapy. 2012;4(9):947–56. 10.2217/imt.12.95 . 23046238

[13] Cao A, Galanello R, Rosatelli MC. Prenatal diagnosis and screening of the haemoglobinopathies. Baillieres Clin Haematol. 1998;11(1):215–38. 10.1016/s0950-3536(98)80076-0 . 10872479

[14] Ioannides AS. Preconception and prenatal genetic counselling. Best Pract Res Clin Obstet Gynaecol. 2017;42:2–10. 10.1016/j.bpobgyn.2017.04.003 . 28533154

[15] Traeger-Synodinos J, Harteveld CL. Preconception carrier screening and prenatal diagnosis in thalassemia and hemoglobinopathies: challenges and future perspectives. Expert Rev Mol Diagn. 2017;17(3):281–91. 10.1080/14737159.2017.1285701 . 28110577

[16] Limwongse C. Medical genetic services in a developing country: lesson from Thailand. Curr Opin Pediatr. 2017;29(6):634–9. 10.1097/MOP.0000000000000544 . 28922317

[17] Kuliev A, Rechitsky S, Verlinsky O, Ivakhnenko V, Cieslak J, Evsikov S, et al Birth of healthy children after preimplantation diagnosis of thalassemias. J Assist Reprod Genet. 1999;16(4):207–11. Epub 1999/05/04. 10.1023/A:1020316924064 . 10224564 PMC3455758

[18] De Rycke M, Van de Velde H, Sermon K, Lissens W, De Vos A, Vandervorst M, et al Preimplantation genetic diagnosis for sickle-cell anemia and for beta-thalassemia. Prenat Diagn. 2001;21(3):214–22. Epub 2001/03/22. 10.1002/1097-0223(200103)21:3<214::aid-pd51>3.0.co;2-4 . 11260611

[19] Chamayou S, Alecci C, Ragolia C, Giambona A, Siciliano S, Maggio A, et al Successful application of preimplantation genetic diagnosis for beta-thalassaemia and sickle cell anaemia in Italy. Hum Reprod. 2002;17(5):1158–65. Epub 2002/05/01. 10.1093/humrep/17.5.1158 . 11980733

[20] Jiao Z, Zhou C, Li J, Shu Y, Liang X, Zhang M, et al Birth of healthy children after preimplantation diagnosis of beta-thalassemia by whole-genome amplification. Prenat Diagn. 2003;23(8):646–51. Epub 2003/08/13. 10.1002/pd.659 . 12913871

[21] Kokkali G, Traeger-Synodinos J, Vrettou C, Stavrou D, Jones GM, Cram DS, et al Blastocyst biopsy versus cleavage stage biopsy and blastocyst transfer for preimplantation genetic diagnosis of beta-thalassaemia: a pilot study. Hum Reprod. 2007;22(5):1443–9. Epub 2007/01/31. 10.1093/humrep/del506 . 17261575

[22] Palmer GA, Traeger-Synodinos J, Davies S, Tzetis M, Vrettou C, Mastrominas M, et al Pregnancies following blastocyst stage transfer in PGD cycles at risk for beta-thalassaemic haemoglobinopathies. Hum Reprod. 2002;17(1):25–31. Epub 2002/01/05. 10.1093/humrep/17.1.25 . 11756357

[23] Kuliev A, Rechitsky S, Verlinsky O, Ivakhnenko V, Evsikov S, Wolf G, et al Preimplantation diagnosis of thalassemias. J Assist Reprod Genet. 1998;15(5):219–25. Epub 1998/05/30. 10.1023/A:1022571822585 . 9604751 PMC3454749

[24] Thornhill AR, deDie-Smulders CE, Geraedts JP, Harper JC, Harton GL, Lavery SA, et al ESHRE PGD Consortium 'Best practice guidelines for clinical preimplantation genetic diagnosis (PGD) and preimplantation genetic screening (PGS)'. Hum Reprod. 2005;20(1):35–48. Epub 2004/11/13. 10.1093/humrep/deh579 . 15539444

[25] Girardet A, Viart V, Plaza S, Daina G, De Rycke M, Des Georges M, et al The improvement of the best practice guidelines for preimplantation genetic diagnosis of cystic fibrosis: toward an international consensus. Eur J Hum Genet. 2016;24(4):469–78. 10.1038/ejhg.2015.99 . 26014425 PMC4929885

[26] Trachoo O, Satirapod C, Panthan B, Sukprasert M, Charoenyingwattana A, Chantratita W, et al First successful trial of preimplantation genetic diagnosis for pantothenate kinase-associated neurodegeneration. J Assist Reprod Genet. 2017;34(1):109–16. 10.1007/s10815-016-0833-y . 27815806 PMC5330985

[27] Griffin DK, Ogur C. Chromosomal analysis in IVF: just how useful is it? Reproduction. 2018;156(1):F29–F50. 10.1530/REP-17-0683 . 29945889

[28] Tongsong T, Charoenkwan P, Sirivatanapa P, Wanapirak C, Piyamongkol W, Sirichotiyakul S, et al Effectiveness of the model for prenatal control of severe thalassemia. Prenat Diagn. 2013;33(5):477–83. 10.1002/pd.4095 . 23553531

[29] Yamsri S, Sanchaisuriya K, Fucharoen G, Sae-Ung N, Ratanasiri T, Fucharoen S. Prevention of severe thalassemia in northeast Thailand: 16 years of experience at a single university center. Prenat Diagn. 2010;30(6):540–6. 10.1002/pd.2514 . 20509153

[30] Corveleyn A, Morris MA, Dequeker E, Sermon K, Davies JL, Antinolo G, et al Provision and quality assurance of preimplantation genetic diagnosis in Europe. Eur J Hum Genet. 2008;16(3):290–9. 10.1038/sj.ejhg.5201976 . 18091772

[31] Harton GL, De Rycke M, Fiorentino F, Moutou C, SenGupta S, Traeger-Synodinos J, et al ESHRE PGD consortium best practice guidelines for amplification-based PGD. Hum Reprod. 2011;26(1):33–40. Epub 2010/10/23. 10.1093/humrep/deq231 . 20966462

[32] Ben-Nagi J, Wells D, Doye K, Loutradi K, Exeter H, Drew E, et al Karyomapping: a single centre's experience from application of methodology to ongoing pregnancy and live-birth rates. Reprod Biomed Online. 2017;35(3):264–71. 10.1016/j.rbmo.2017.06.004 . 28648921

[33] Gould RL, Griffin DK. Karyomapping and how is it improving preimplantation genetics? Expert Rev Mol Diagn. 2017;17(6):611–21. 10.1080/14737159.2017.1325736 . 28459185

[34] Kubikova N, Babariya D, Sarasa J, Spath K, Alfarawati S, Wells D. Clinical application of a protocol based on universal next-generation sequencing for the diagnosis of beta-thalassaemia and sickle cell anaemia in preimplantation embryos. Reprod Biomed Online. 2018;37(2):136–44. 10.1016/j.rbmo.2018.05.005 . 29853423

[35] Smith WM, Van Orsouw NJ, Fox EA, Kolodner RD, Vijg J, Eng C. Accurate, high-throughput "snapshot" detection of hMLH1 mutations by two-dimensional DNA electrophoresis. Genet Test. 1998;2(1):43–53. Epub 1999/08/28. 10.1089/gte.1998.2.43 . 10464596

[36] Konkle BA, Huston H, Nakaya Fletcher S. Hemophilia A In: Adam MP, Ardinger HH, Pagon RA, Wallace SE, Bean LJH, Stephens K, et al, editors. GeneReviews((R)). Seattle (WA) 1993.

[37] Darras BT, Urion DK, Ghosh PS. Dystrophinopathies In: Adam MP, Ardinger HH, Pagon RA, Wallace SE, Bean LJH, Stephens K, et al, editors. GeneReviews((R)). Seattle (WA) 1993.

[38] Demko ZP, Simon AL, McCoy RC, Petrov DA, Rabinowitz M. Effects of maternal age on euploidy rates in a large cohort of embryos analyzed with 24-chromosome single-nucleotide polymorphism-based preimplantation genetic screening. Fertil Steril. 2016;105(5):1307–13. 10.1016/j.fertnstert.2016.01.025 . 26868992

[39] Chiamchanya C, Visutakul P, Gumnarai N, Su-angkawatin W. Preimplantation genetic screening (PGS) in infertile female age > or = 35 years by fluorescence in situ hybridization of chromosome 13, 18, 21, X and Y. J Med Assoc Thai. 2008;91(11):1644–50. . 19127783

[40] Friedenthal J, Maxwell SM, Munne S, Kramer Y, McCulloh DH, McCaffrey C, et al Next generation sequencing for preimplantation genetic screening improves pregnancy outcomes compared with array comparative genomic hybridization in single thawed euploid embryo transfer cycles. Fertil Steril. 2018;109(4):627–32. 10.1016/j.fertnstert.2017.12.017 . 29605407

[41] Cimadomo D, Fabozzi G, Vaiarelli A, Ubaldi N, Ubaldi FM, Rienzi L. Impact of Maternal Age on Oocyte and Embryo Competence. Front Endocrinol (Lausanne). 2018;9:327 10.3389/fendo.2018.00327 . 30008696 PMC6033961

[42] Lamb B, Johnson E, Francis L, Fagan M, Riches N, Canada I, et al Pre-implantation genetic testing: decisional factors to accept or decline among in vitro fertilization patients. J Assist Reprod Genet. 2018;35(9):1605–12. 10.1007/s10815-018-1278-2 . 30074131 PMC6133803

[43] Fritz R, Jindal S. Reproductive aging and elective fertility preservation. J Ovarian Res. 2018;11(1):66 10.1186/s13048-018-0438-4 . 30098598 PMC6087539

[44] Zeng C, Xu JN, Zhou Y, Zhou YF, Zhu SN, Xue Q. Reproductive performance after surgery for endometriosis: predictive value of the revised American Fertility Society classification and the endometriosis fertility index. Gynecol Obstet Invest. 2014;77(3):180–5. 10.1159/000358390 . 24603632

[45] Seyhan A, Ata B, Uncu G. The Impact of Endometriosis and Its Treatment on Ovarian Reserve. Semin Reprod Med. 2015;33(6):422–8. 10.1055/s-0035-1567820 . 26594869

[46] Ozekinci M, Seven A, Olgan S, Sakinci M, Keskin U, Akar ME, et al Does obesity have detrimental effects on IVF treatment outcomes? BMC Womens Health. 2015;15:61 10.1186/s12905-015-0223-0 . 26285703 PMC4543460

[47] Kekre N, Antin JH. Hematopoietic stem cell transplantation donor sources in the 21st century: choosing the ideal donor when a perfect match does not exist. Blood. 2014;124(3):334–43. 10.1182/blood-2014-02-514760 . 24914138

[48] Rafati M, Akhondi MM, Sadeghi MR, Tara SZ, Ghaffari SR. Preimplantation High-Resolution HLA Sequencing Using Next Generation Sequencing. Biol Blood Marrow Transplant. 2018;24(8):1575–80. 10.1016/j.bbmt.2018.03.024 . 29649618

